# Event-driven proto-object based saliency in 3D space to attract a robot’s attention

**DOI:** 10.1038/s41598-022-11723-6

**Published:** 2022-05-10

**Authors:** Suman Ghosh, Giulia D’Angelo, Arren Glover, Massimiliano Iacono, Ernst Niebur, Chiara Bartolozzi

**Affiliations:** 1 Event Driven Perception for Robotics, Istituto Italiano di Tecnologia, 16163 Genoa, Italy; 2 Department of Computer Science, The University of Manchester, Manchester, M13 9PL UK; 3 Mind/Brain Institute, Johns Hopkins University, Baltimore 21218 MD, USA; 4 Present Address: Electrical Engineering and Computer Science, Technische Universität Berlin, 10623 Berlin, Germany

**Keywords:** Electrical and electronic engineering, Computer science, Attention

## Abstract

To interact with its environment, a robot working in 3D space needs to organise its visual input in terms of objects or their perceptual precursors, proto-objects. Among other visual cues, depth is a submodality used to direct attention to visual features and objects. Current depth-based proto-object attention models have been implemented for standard RGB-D cameras that produce synchronous frames. In contrast, event cameras are neuromorphic sensors that loosely mimic the function of the human retina by asynchronously encoding per-pixel brightness changes at very high temporal resolution, thereby providing advantages like high dynamic range, efficiency (thanks to their high degree of signal compression), and low latency. We propose a bio-inspired bottom-up attention model that exploits event-driven sensing to generate depth-based saliency maps that allow a robot to interact with complex visual input. We use event-cameras mounted in the eyes of the iCub humanoid robot to directly extract edge, disparity and motion information. Real-world experiments demonstrate that our system robustly selects salient objects near the robot in the presence of clutter and dynamic scene changes, for the benefit of downstream applications like object segmentation, tracking and robot interaction with external objects.

## Introduction

Every agent, whether animal or robotic, needs to process its sensory input in an efficient way, to allow understanding of, and interaction with, the environment. Since the agent’s computational capabilities are limited, careful allocation of perceptual and cognitive resources is required^1^. The process of filtering relevant information out of the continuous bombardment of complex sensory data is called selective attention. This process not only occurs in animals, where the selection of the most ecologically important stimuli like the presence of a predator is required but also in complex machinery with a rich array of sensors, like robots. The large amount of information arriving in the information processing stages at all times from sensors that are needed only at some times cannot be processed economically in its entirety. Selective attention mechanisms are used to analyse only the most important subset of the sensory stream. A number of visual attention algorithms have been proposed in robotics exploiting selective attention mechanisms^2–6^.

**Figure 1:**
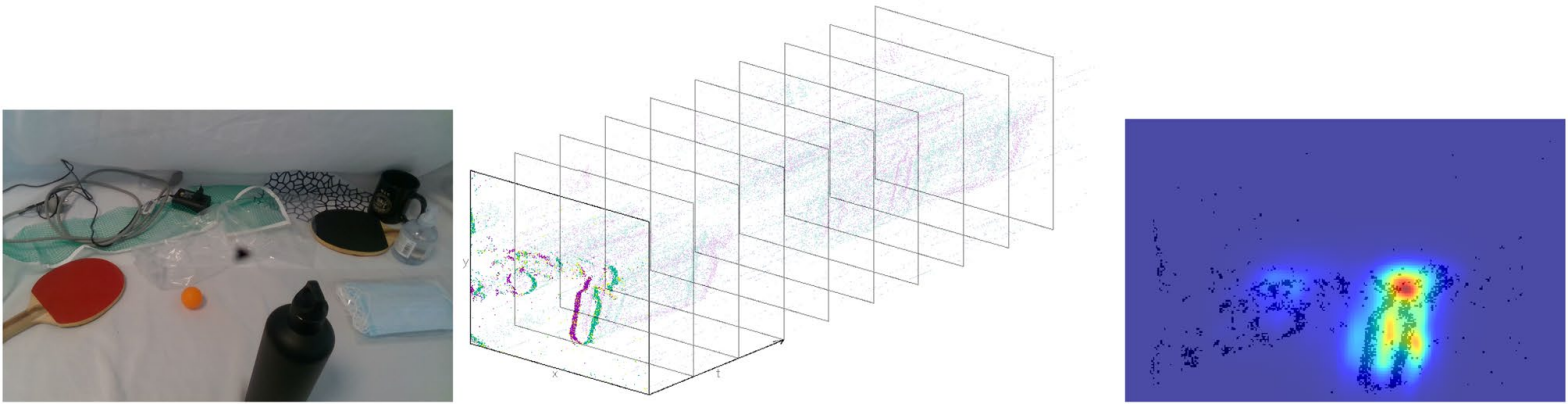
Event-driven proto-object saliency estimation in 3D. Left: Cluttered table top with objects of different sizes and textures placed at varying depths (only for visualisation). Middle: Events produced from the ATIS camera using circular robot eye motion. The event stream is plotted in spatio-temporal coordinates. The green and purple colours represent whether the pixel witnessed a brightness increase or decrease. Events from the stereo cameras serve as input to our model. Right: Saliency map computed using the proposed evProtoDepth model, with the closest object (black bottle) selected. The saliency map is overlaid on the event image generated by accumulating events generated within a 100 ms time window.

Visual attention is the result of the complex interplay between the physical characteristics of the scene (stimulus-driven, bottom-up mechanisms) and the goals of the agent (task-dependent, top-down mechanisms)^7^. Bottom-up models of selective attention rely both on feature extraction^8–10^ and perceptual organisation of the scene^11^. Mechanisms of perceptual organisation have been formalised in the form of “Gestalt laws” (e.g. continuity, proximity, figure-ground segmentation) that contribute to the grouping of visual features into coherent objects^12^. These principles can be integrated into feature based bottom-up models^13,14^ to identify so-called proto-objects^11^, by adding a layer of Gabor^8^, or curved Von Mises filters^11^, loosely similar to neuronal responses in primate visual cortex^15^. Such models use biological inspiration by emulating the cells that extract visual features and combine them using border ownership and grouping mechanisms, to produce a robust saliency map of the scene that increases perceptual saliency of regions with object-like stimuli.

We are interested in the bridge between biologically plausible models, bio-inspired hardware, and embodied agents (robots) to further understand the role of the hardware and the environment in selective attention processes. Our previous work^16^ implemented the proto-object model proposed by Russell et.al.^11^ using bio-inspired artificial visual sensors, called event-driven cameras^17^. The event-driven cameras function more similarly to biological eyes than frame-based cameras. Instead of scanning each pixel in order to measure the incident light level as in a traditional camera, each pixel in an event camera is independent and produces a spike when the incident light changes beyond a threshold. These “pixel spikes” are similar in function to the action potentials that the retina sends to the brain. The output of the event-camera is asynchronous, sparse, and occurs only where there is a differential between dark and light regions of the scene detected as an illumination change of each pixel over time, functioning *de facto* as a dynamic edge extractor. The integration of the event-camera into the proto-object processing pipeline inherently performs some of the lower-level processing that the model requires (detecting illumination change), opening interesting questions on the role of the hardware, as well as the brain, in sensory processing.

**Figure 2:**
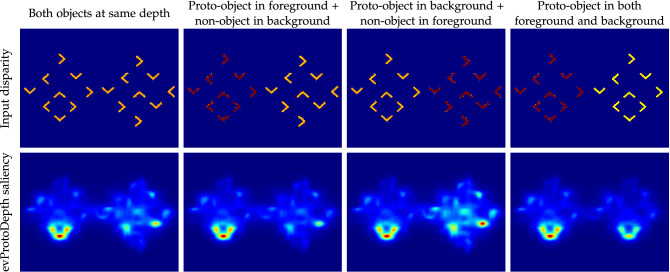
Interplay between depth (disparity) and Gestalt cues in evProtoDepth saliency. The disparity maps (Row 1) have two possible depths: near (dark red) and far (light orange), and the evProtoDepth saliency (Row 2) is shown from strong (red) to weak (blue). Arranging the angle features in a convex shape generates a perceptual (proto-)object that contributes to saliency in our model. Turning any of the angles in a different orientation destroys object perception. This contribution to saliency is integrated with that resulting from differences in depth. The salience of the synthetic proto-object pattern increases as it moves closer to the camera. However, even when the proto-object moves further away in the background, it produces a strong response compared to the non-object pattern in the foreground. This demonstrates the advantage of using a proto-object model instead of directly relying on raw scene depth for nearest “object” selection by the robot. The selectivity is the strongest when the proto-object is placed closer to the camera while the non-object pattern is in the background (Column 2).

Relative depth and apparent object size provide important cues to guide bottom-up attention mechanisms during physical scene interpretation^18–20^. Depth cues from binocular disparity have been shown to modify eye movements of participants when shown 3D images^21^ and videos^22^. Directed attention to local features have also been shown to aid in the interpretation of three-dimensional cues^23^. To explore the role of depth in event-driven attention, in this paper, we extend our previously developed event-driven proto-object model (evProto)^16^ by combining it with a biologically inspired stereo disparity estimation algorithm^24^, resulting in a depth-based attention model. Furthermore, our implementation runs online on a robotic platform (the neuromorphic iCub^25^).

In two previous studies, a proto-object based model of selective attention^11^ was extended to include depth in the saliency map computation^26,27^. Our model goes beyond those studies mainly in two ways. First, both of these models are frame-based while we use input from neuromorphic event cameras. Second, both models require supplementary information in addition to the two input images. A full depth map obtained by an RGB-D sensor is needed for the Hu et al. model^26^. The Mancinelli et al. model^27^ does obtain depth information from stereoscopic cameras but it assumes that a certain number of known correspondence points are available. Instead, our model solves the correspondence problem directly, using only visual input streams from two event-driven cameras by making use of the precise signal timing at the pixel level, as is described in Methods.).

An important concept for all agents interacting with their physical environment, be it humans, animals or robots, is the implicit, underlying interpretation of the environment imposed by object affordance^28^; the object features that define their possible uses and/or make clear how they can or should be used^29^. It seems reasonable to expect that there is a bi-directional relationship between affordances and salience: Affordances are important for interacting with objects, so they need to be attended to make this interaction possible. On the other hand, features related to affordances may be salient by themselves, either by their inherent visual properties (shape etc.) or by their design (e.g. painting a handle red). There is evidence for a bidirectional relationship between attention and affordance^30,31^, while other studies have shown that the correlation may not be particularly strong^32–34^. The relationship may be more nuanced and be affected by additional neurological systems, which would require additional study. We note, however, that even though we do not include any explicit consideration of affordances in our study, we direct the robot’s attention towards objects in a certain size range, according to its grasping capabilities^16^. Furthermore, in our implementation of the depth channel we increase the saliency of closer objects, which are therefore easier to reach by the robot, which is an affordance of elementary importance.

Our motivation is to understand the benefits of combining biologically inspired algorithms with neuromorphic hardware on embodied agents, as opposed to improving the precision and performance of the object selection or eye fixation prediction. The objective we pursue with our attention model is to produce saliency maps that are robust to noise, quickly adapt to dynamic changes in the visual scene, and remain close to important biological processing mechanisms. As the system produces saliency estimation using event-driven cameras based on depth information, we will refer to it as the evProtoDepth (event-driven Proto-object 3D) model. It is able to cope both with dynamic scenes (with motion) and with static images. In order to process the latter, small periodic stereotyped ocular movements are performed by the robot to generate stimulus dependent activity from event-driven cameras to generate pixel motion, akin to microsaccades in biological vision^35^.

Since we want the robot to be more attentive to nearby objects that are within its reach, our saliency model design puts a higher importance on stimuli with higher disparity. This allows nearby objects to inherently appear more salient. Besides the affordance of reachability, our design choice is also based on ecological evidence which suggests that attention in insects, mice and humans is drawn towards looming stimuli^36–38^, wherein nearby approaching objects are deemed especially important. Whereas other features also contribute to salience in full attention systems, here we focus on depth alone and leave the integration with other submodalities for future work. Thus, the evProtoDepth model selects the nearest potential object (proto-object) that the robot could reach and interact with as the most important item in the scene (see Fig. 1). To fully explore the influence of depth on the event-driven saliency model, we propose a depth-only implementation as the base for a more complex saliency based attention system in the future, in which multiple features are weighted based on top-down mechanisms to adapt the detection of salient regions of the scene to the task at hand^39^.

In the next section, we demonstrate the performance and suitability of the event-driven stereo depth algorithm as an input to the proposed attention model. A comparison of the proposed evProtoDepth and the non-event-based proto-object attention model is made on publicly available attention-based datasets, and a series of tests on the iCub robot are made to demonstrate the attention to nearby objects, as opposed to nearby non-objects and far away objects.

**Table 1:** Consolidated MIT saliency metrics Normalized Scanpath Saliency (NSS), Area under the ROC Curve (AUC-Borji), Kullback–Leibler Divergence (KLDiv), Pearson’s Correlation Coefficent (CC) and Similarity (SIM)^40–43^ on the closest-object subset of the NUS3D dataset. A higher score is better for all metrics, excluding the KLDiv. Bold font indicates the model with the better performance. Some of the corresponding scenes and saliency maps are depicted in Fig. 3. The metrics for each individual image in this subset are presented in Supplementary Fig. S8.

	NSS	AUC_Borji	KLDiv	CC	SIM
	Mean, median	Mean, median	Mean, median	Mean, median	Mean, median
fbProtoDepth^26^	**0.936**, **0.917**	**0.737**, **0.747**	**1.603**, **1.501**	0.386, 0.386	0.283, 0.296
evProtoDepth	0.769, 0.888	0.606, 0.592	2.971, 2.249	**0.417**, **0.417**	**0.401**, **0.420**

**Figure 3:**
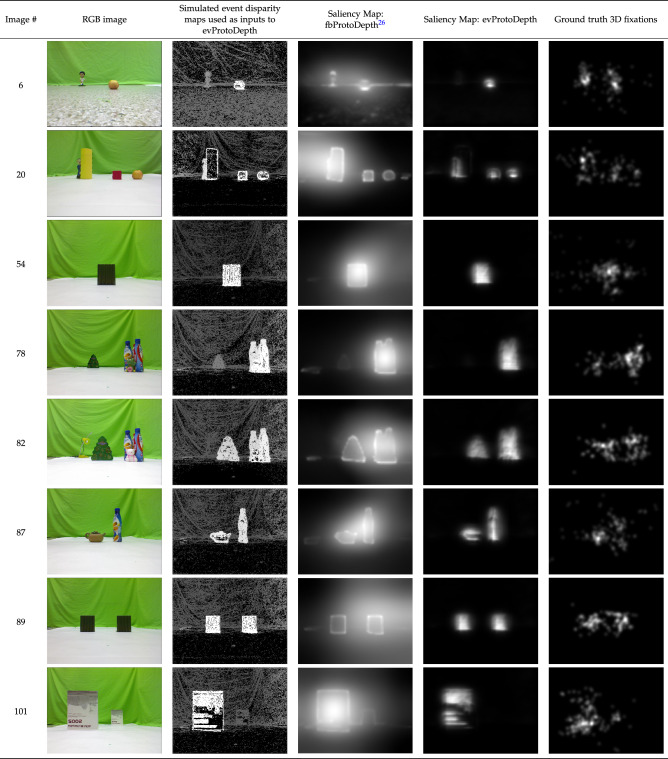
Comparison of saliency maps generated by fbProtoDepth^26^ and evProtoDepth on samples from a subset of the NUS3D dataset where ground truth fixation was concentrated on the nearest object in the scene. The subset comprises all cases where the cross-correlation between the ground truth 3D fixation and inverse of ground truth depth $\ge 0.5$. These scenes depict scenarios relevant to a robot application where the goal is to select the nearest “object”. This benchmarking experiment investigates how depth contributes to event-based proto-object saliency for predicting human eye fixations in such scenarios. evProtoDepth uses a single depth channel for saliency prediction whereas fbProtoDepth combines information from parallel depth, colour opponency, intensity and orientation channels at the final stage. This causes the former to generate sparser saliency maps highly localised on the nearest object which are suitable for robot applications like segmentation and grasping.

## Results

The evProtoDepth model is biased to select the closest object in the scene, and to decrease the saliency of near stimuli that do not fulfil the continuity and proximity conditions that define the presence of a proto-object, as shown in Fig. 2. We evaluate the evProtoDepth model against the standard frame-based proto-object model (fbProtoDepth)^26^ on a subset of the NUS3D publicly available dataset^44^, comparing also to ground truth fixation maps captured from human eye tracking data. We validate the accuracy of the on-line event-based depth estimation model and on the neuromorphic iCub robot^25^ with live visual data from stereo ATIS cameras^17^, and evaluate the response of the full evProtoDepth pipeline on the iCub robot to identify salient regions produced by nearby objects in the scene.

The model takes $\approx 170 \; \hbox {ms}$ to compute saliency of one frame on a laptop with Nvidia GTX 1650 GPU and Intel Core i7-9750H CPU @ 2.60 GHz $\times$ 12. The parameters used to run the model are specified in the supplementary material (Supplementary Tables S1 and S2). An accompanying video (https://zenodo.org/record/5091539) supports an intuitive understanding of the experiments.

### Saliency benchmarking with NUS3D saliency dataset

The NUS3D dataset ^44^ is used to quantitatively compare event-based evProtoDepth with frame-based fbProtoDepth against a ground-truth saliency map. The goal of the analysis is to understand how close we are to the real fixation maps in cases where humans fixate mostly on the closest object. To this aim, we algorithmically selected a subset of **19** images from the dataset in which the highest salient region should be the closest object, i.e. images in which the cross-correlation between the ground truth fixation map and the inverse of ground truth depth is $\ge \; 0.5$.

The dataset provides colour RGB input stimuli, depth maps as well as locations of fixations when humans fixated on either the 2D or 3D images. To produce simulated “micro-saccades” (see above), the still images were shifted by 1 pixel in the cardinal directions (right, left, top and bottom) to simulate random small eye motion^45^ and a video of 50 frames (25 fps) was created for each input image. Events were generated from the video using the Open Event Camera Simulator^46^. Depth was assigned to each event using the ground-truth depth map for each pixel and smoothed by 1 pixel in each direction to account for the eye-motion. The evProtoDepth saliency map is computed from the simulated events whereas the fbProtoDepth is computed from the static RGB and depth images in the dataset. Fig. 3 shows that both models detect the objects in the scene focusing the attention on the closest one. The fbProtoDepth shows a wider and centre-biased response, whereas the evProtoDepth shows a more localised response which is useful in a robotic context. It allows the robot to pinpoint the location of most salient parts of the scene with higher precision and confidence, which is important for subsequent physical interaction.

The Normalized Scanpath Saliency (NSS), Area under the ROC Curve (AUC-Borji), Kullback-Leibler Divergence (KLDiv), Pearson’s Correlation Coefficient (CC) and Similarity (SIM) are computed as metrics to compare the saliency maps to the ground-truth, following standard analysis methods in the literature^40–43^. A single saliency map cannot perform well in all the metrics since they judge different aspects of the similarity between ground truth and predicted saliency map^47^.

The fbProtoDepth model has better performance than evProtoDepth on three of the five metrics (NSS, AUC-Borji, and KLDiv), while evProtoDepth achieves a better result for the CC and SIM metrics as shown in Table 1. The fbProtoDepth model uses intensity, colour, and opponency channels, while evProtoDepth uses only the depth channel, and as such saliency patterns are not expected to be identical between methods.

The response of both models and the ground truth all peak on the closest objects, as shown in Fig. 3. While there is not a large amount of clutter in the dataset, it is clear from the second column of Fig. 3 that the intensity gradient of the background (curtain) is non-negligible and produces many background events. The signal from the background does not conform to the proto-object pattern and therefore is correctly suppressed by the models. In cases in which there is a large difference between the object depth, all models successfully produce a stronger response to the closest object.

**Figure 4:**
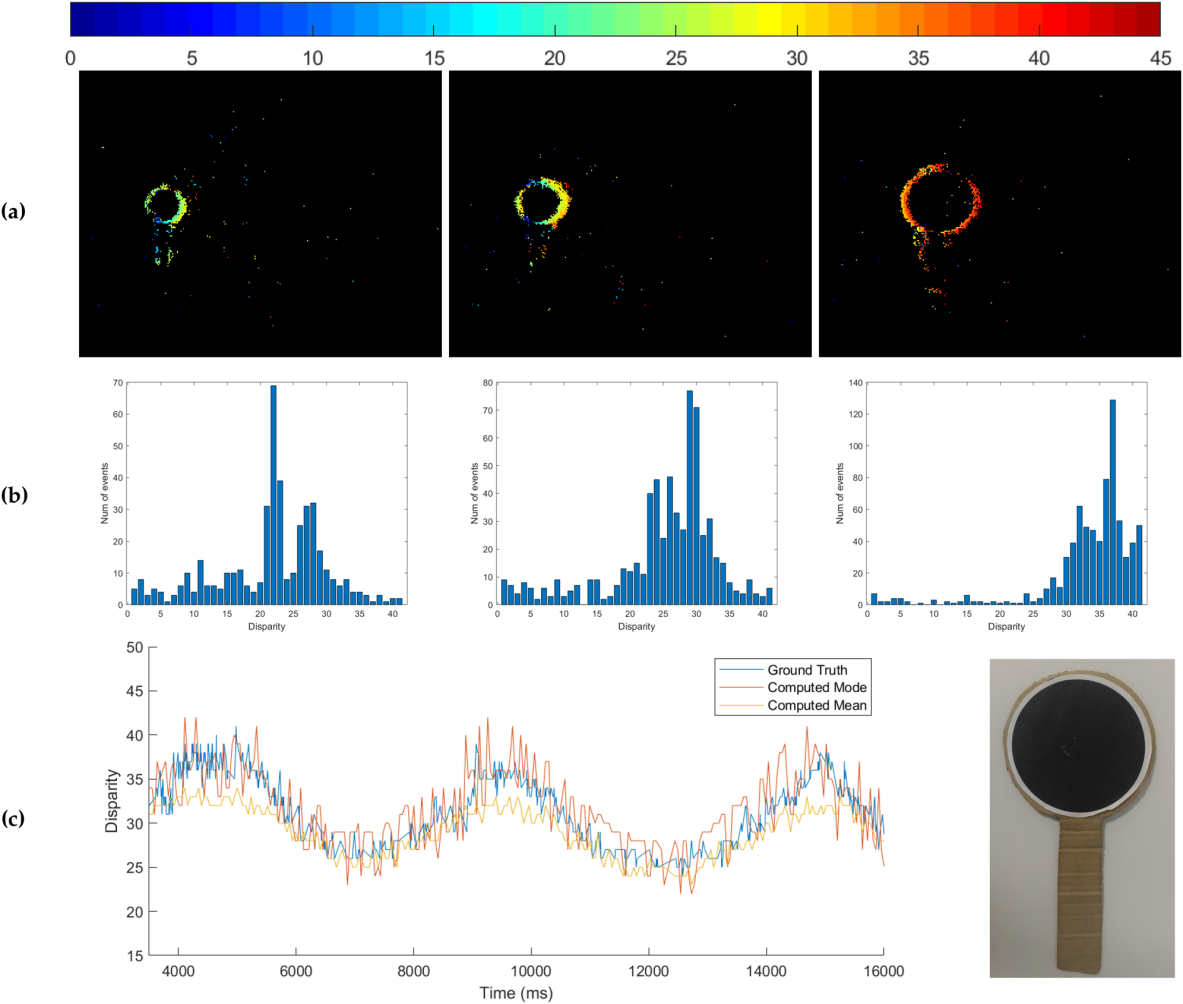
Evaluation of estimated disparity accuracy of a circular paddle moving to and fro along the depth axis. (**a**) Colour-coded (red = near, blue = far) event disparity maps (time window 100 ms) of the paddle at three time instances: far (left), intermediate (centre) and close (right). (**b**) The corresponding disparity distribution histograms. (**c**) Variation of ground truth and computed disparity (mean and mode within manually annotated ROI) over time, and an image of the input stimulus.

Even in scenes where the ground truth 3D eye fixations were not necessarily confined to the nearest “proto-object”, the event-driven evProtoDepth model may produce saliency maps concentrated on the nearest object following Gestalt principles, because it relies on depth information. By contrast, fbProtoDepth, which relies on multiple information channels besides depth, better predicts eye fixations. Some examples of such scenes are shown in Supplementary Fig. S9.

### Disparity estimation for the neuromorphic iCub

The accuracy of the disparity estimation model is demonstrated online (50 microseconds latency per event) on the robot by moving a high-contrast fiducial marker, a circle shape, at different distances (within a 30–210 cm range) from the stereo cameras and comparing the computed disparity to the ground truth. The ground truth is computed by tracking the circle shape^48^ independently in each camera and computing the horizontal distance between the circle centres in the left and right cameras.

The ground truth is compared to the mean and mode of estimated disparity values within a Region of Interest (ROI) placed around the tracked circle centre. Figure 4 shows accuracy of disparity estimation qualitatively and quantitatively. The histogram peaks position in Fig. 4b corresponds to the depth of the stimulus shown in Fig. 4a. Figure 4c shows quantitatively that the estimated disparity is accurate with respect to the ground truth throughout the sequence. The jitter is due to imperfect time correspondence in the asynchronous system.

Further experiments with more complex multi-object stimuli are presented in the supplementary material (Supplementary Fig. S7). We observe that even the noisy disparity map manages to reflect the real scene depth to accurately represent the dynamic environment. The network simultaneously encodes different levels of disparity information, solving the correspondence problem, at different spatial locations and times, consistent with real world depth. The model is capable of resolving the depth of complex stimuli like the human body, with multiple non-rigid moving parts.

### Robot application of 3D proto object model

To validate the evProtoDepth model, we implemented a robot application where iCub uses its movable stereo event-driven cameras to observe static and moving stimuli and selects the nearest proto-object with the goal of further physical interaction. Specifically, we tested whether the evProtoDepth implementation consistently selects the nearest object in the scene as the most salient, when the depth of objects changes dynamically. At the same time, an important aspect of our evaluation is the stability of object selection when the scene configuration remains constant, and the model’s robustness to noise both in the background and foreground.

**Figure 5:**
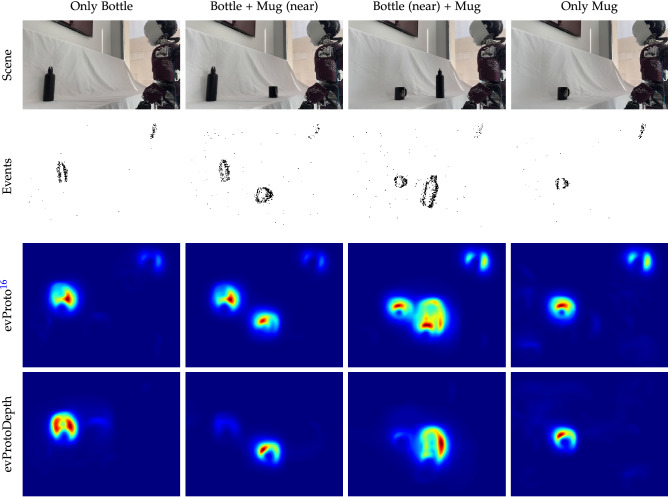
Static objects at changing depth *(bottle-mug)* dataset. The columns show snapshots from the 4 different configurations in the dataset. Row 1 depicts the scene from a third perspective. Row 2 shows the event images accumulated over 100 ms. Row 3 and 4 illustrate the output generated by the evProto and the evProtoDepth models respectively. Each plot shows the 2D histogram of saliency maps accumulated over all frames in a single input configuration. Object selection is more stable with the 3D model in the presence of multiple objects.

**Figure 6:**
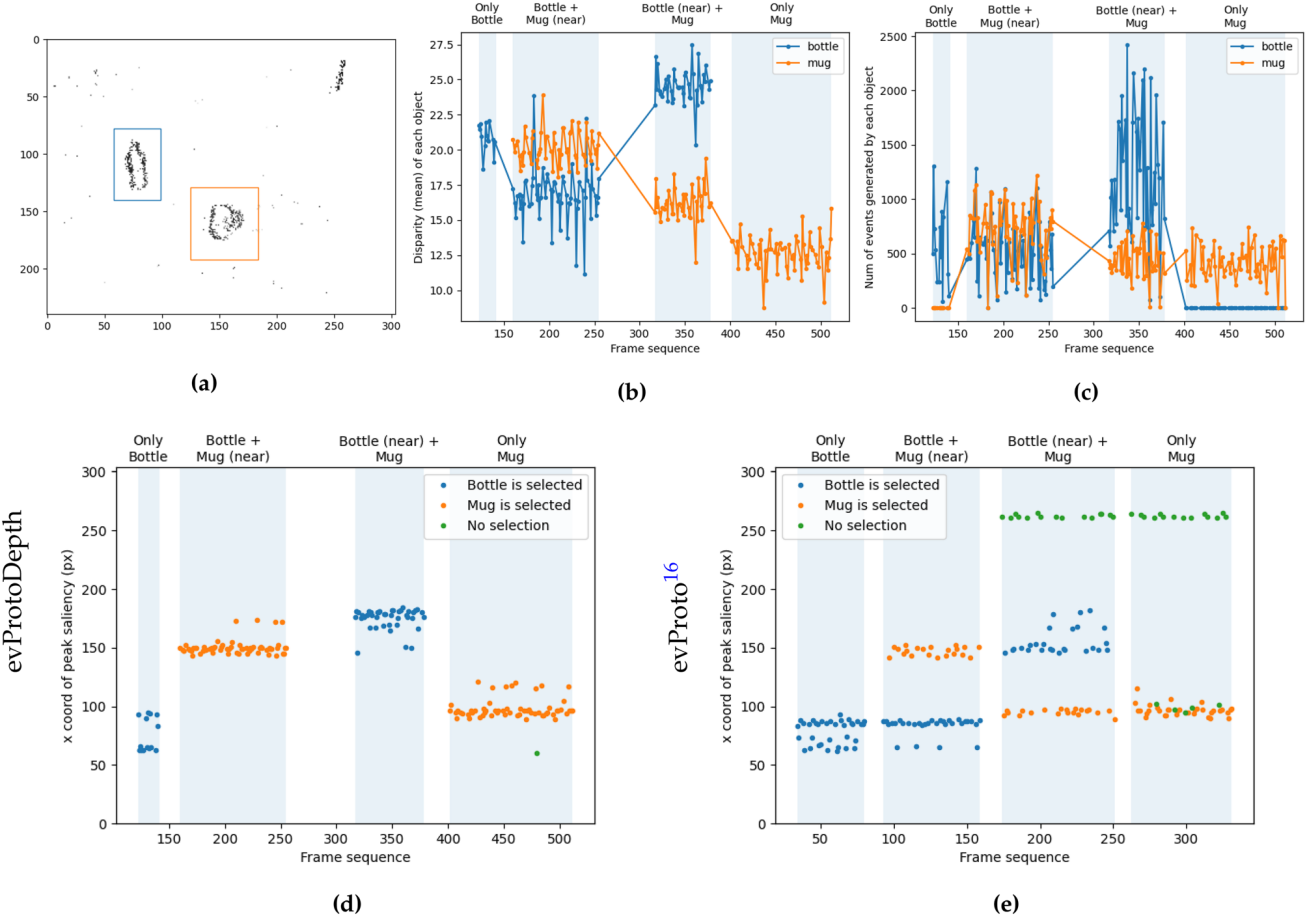
The disparity channel stabilises object selection in the *bottle-mug* dataset, during which the object positions are moved on a table. (**a**) Sample event frame with manually annotated object boundaries—at this particular time, the mug is closer to the camera. (**b**) Mean disparity within each object boundary in both object frames. (**c**) Number of events generated within each object boundary in both event frames. (**d**,**e**) *x* co-ordinates of the peak response in each frame for the evProtoDepth and evProto attention models. For each frame, an object is “selected” if the peak saliency pixel lies within its annotated boundaries, otherwise “No selection” occurs. There is only one unique object selection at each time stamp (frame). This means that for evProto in (**e**), the saliency peak jumps from one object to another frequently. Thus the orange, blue and green dots occur at (different) timestamps very close to each other.

**Figure 7:**
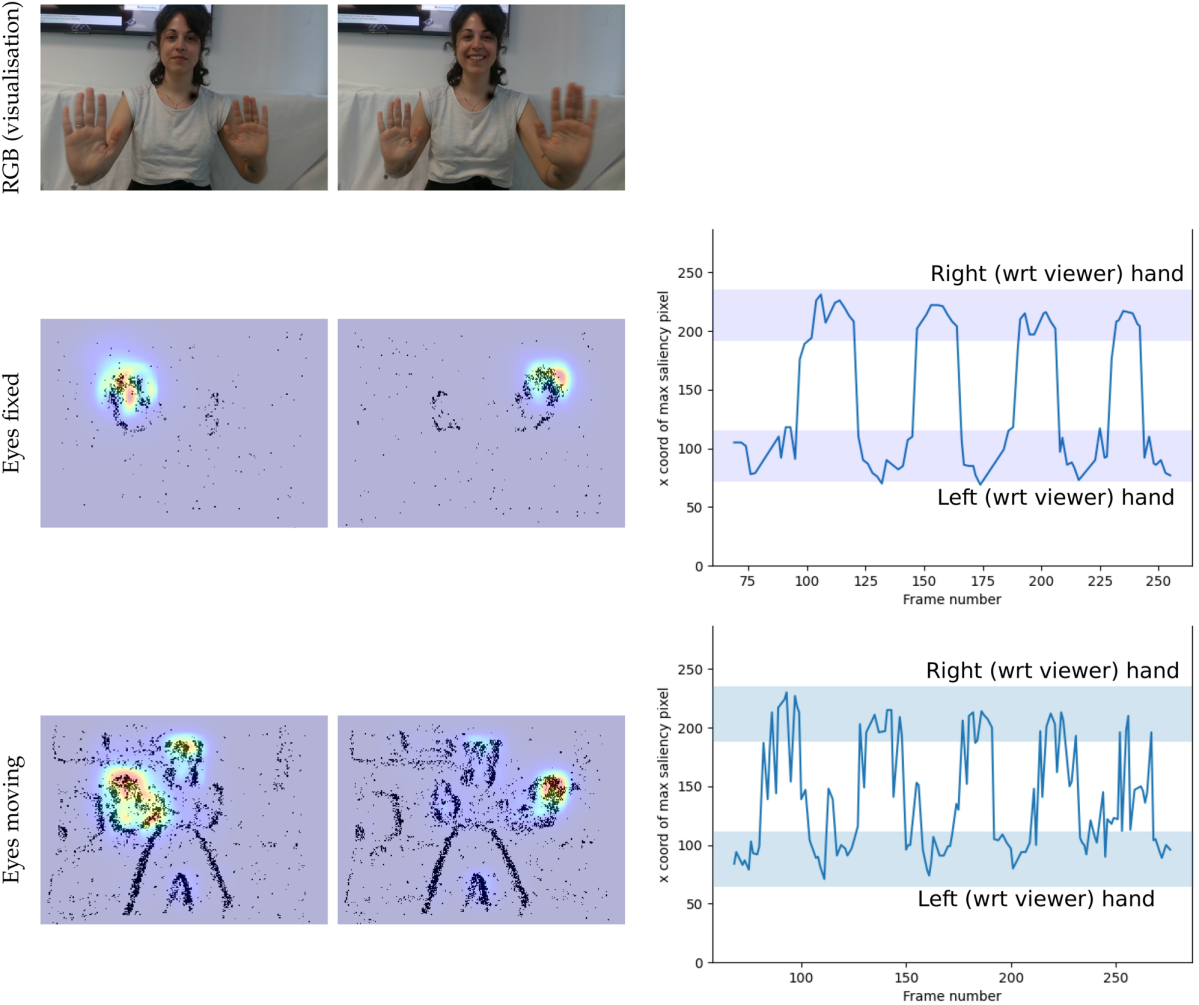
Saliency prediction from evProtoDepth in a dynamic scene (data set *hands*) containing hands moving towards the cameras and away from them, with and without the iCub eye motion. The events from the stereo cameras are the only input to our model. RGB (Row 1) frames at different instances of the sequence are shown for visualisation. The two leftmost columns of Rows 2 and 3 depict corresponding saliency outputs overlaid on input events while the robot eyes were fixed (Row 2), and moving (Row 3). With *fixed eyes*, only the moving hands trigger events, whereas with *moving eyes*, events are generated by static as well as moving features in the scene, thus both static (e.g. the face) and moving objects (hands) appear salient. The rightmost column shows the *x* co-ordinates (along the axis between the person and the cameras) of peak saliency plotted against time (frame number) for both datasets. The true locations of the hands are marked with coloured bands. For *static cameras* (Row 2), the peak saliency pixel consistently alternates between the left and right hand locations as they move towards and away from the camera, i.e., it follows the hand closest to the camera. For *moving eyes*, (Row 3), excess events caused by micro-saccades result in some spurious saliency peaks at objects like the face despite them being farther away from the camera.

Figure 5 shows how the addition of depth information improves object selection stability. The 2D histogram of saliency maps (bottom two rows) obtained during each object configuration shows that both models can select plausible objects in the scene. The addition of the disparity information in the evProtoDepth model, however, enhances the salience of the object which is closer to the observer. While the 2D evProto model assigns overall similar saliency values to the bottle and the mug. The development of saliency over time is shown for both models in Fig. 6. The comparison between Fig. 6d and its evProto counterpart (Fig. 6e) shows that the peak response of the evProto model jumps from one object to the other even when the scene configuration remains unchanged. Furthermore, the peak of the saliency map obtained from the evProto model often occurs outside the annotated object boundaries (green dots, “No selection”). As an example, in Fig. 5 Row 3 shows that the evProto model finds the ray of the sunlight on the top-right corner of the wall (as seen in the colour image in Row 1) as highly salient. Object disparity therefore stabilises object selection (see Fig. 6b). The selection does not depend on the number of events generated by the object, as plotted in Fig. 6c: during the “Bottle + Mug (near)” configuration, the evProtoDepth selects the mug which is closer to the camera, even though both objects generate similar number of events.

The experiment of Fig. 7 investigates the response of the system to continuously changing stimuli, in the example shown, a person alternately moving the left and right hand towards and away from the iCub. The location of attention quickly and reliably shifts to the nearest proto-object as soon as the relative position of the hands change sign. The rightmost column shows the location of maximum salience over time, confirming the switch of attention from the left hand to the right one while the hands were moving, even when the eyes of the robot are moving. In this second scenario, events are generated by the moving cameras from static objects, leading to high saliency at intermediate depth locations as well (e.g. the face of the person standing in front of the camera). However, most of the time the closest objects are selected. This experiment demonstrates that the evProtoDepth model can in real-time track the closest object in a dynamic scene with eyes fixed and in motion.

To obtain a fair comparison between our implementation and the fbProtoDepth model, we recorded RGB-D frames from a Real-Sense D435 depth camera that uses active IR stereo technology to record depth information along with visual images. The depth maps were post-processed with hole-filling filters provided in the Real-Sense library. These holes are 0 value pixels which would otherwise be erroneously treated as the nearest stimuli by the attention models.

The direct comparison between the evProtoDepth and the fbProtoDepth qualitatively on *hands* dataset depicts that the fbProtoDepth shows a wider and centre-biased response, whereas the evProtoDepth shows a more localised response because event-driven cameras only respond to motion and high contrast changes and generate sparse features. The fbProtoDepth takes the entire human as single object due to the presence of additional orientation and colour opponency channels, whereas in case of evProtoDepth, the event cameras produce sparse and disjointed features leading to the detection of multiple smaller objects. This can be observed in Supplementary Fig. S10.

The evProtoDepth model is able to focus the attention towards the target which is closer to the robot, making it more suitable for behavioural decisions and interaction within its proximity. The system shows reliable response in cluttered scenarios and dynamic scenes. The Disparity Extractor alone provides a disparity map without any higher level filtering of “objects” in the scene. Therefore, the integration of the evProto model with the disparity extractor informs the system about salient regions which are not only nearby but also follow Gestalt laws. The proto-object model helps select a proto-object following Gestalt laws while discarding noise from the disparity map, whereas the additional disparity information improves selection precision in evProtoDepth. For evidence we point the reader to the Supplementary Fig. S11 which depicts 2D histograms of peak responses for evProto, Disparity and evProtoDepth saliency maps.

## Discussion

We introduce a model that combines disparity computations based on neuromorphic event-driven algorithms and hardware with a bio-inspired attention model. It improves upon the 2D model (the evProto model) which assigns perceptual saliency to (moving) edges that enclose a region (not necessarily completely) and can hence form the contour of an object. Adding the disparity information results in our 3D evProtoDepth model which, in addition to the salience imparted by evProto, assigns additional saliency to regions that are also closer to the cameras compared to those at larger distance. Adding depth information provides more stable object selection and robustness to noise, as demonstrated in Figs. 5 and  6.

From the results presented about disparity estimation (see Fig. 4 and Supplementary Fig. S7), the event-based disparity estimation is robust and reliable in different scenarios with dynamic objects of increasing complexity. It can solve the correspondence problem for multiple objects simultaneously, distinguishing their relative distance from the robot. When the stream of events increases because of clutter and/or eyes movements, the accuracy of the disparity estimation is traded-off with latency, increasing the level of noise. Typically, the disparity information successfully enables the attentive system to select the nearest proto-object. The online evaluation implemented on a robot using real-world data proves the capabilities of the model in a realistic scenario. The system is robust to clutter and it demonstrates robust selection of the nearest proto-object in a noisy background. The robot is responsive to motion, giving preference to closer moving objects. When we enable eyes motion, it can also select the nearby static object. The model tolerates motion of the cameras and of scene objects and usually determines as salient those areas that are closest to the cameras. The use of a biologically inspired event-driven disparity extractor distinguishes the evProtoDepth model from its frame-based counterpart fbProtoDepth. While the latter requires a pre-computed depth map from RGB-D sensor and computes feature maps representing local intensity, colour opponency and orientations, the only input required by our new evProtoDepth are raw streams of events from two neuromorphic cameras. Disparity information is extracted directly from these event streams using a bio-inspired cooperative matching algorithm. Benchmarking on the NUS3D dataset shows that despite those differences both models achieve similar performance, with the event-driven one being more easily applicable to online robotic applications, thanks to a more localised response over the selected objects.

Both models, fbProtoDepth and evProtoDepth, have strict bottom-up (data-driven) architectures and achieve mediocre results on the MIT metrics when directly compared with the eye fixation maps. This is expected due to the presence of complex attention mechanisms which include influences that are not captured by either of the models. These influences include cognitive top-down (goal driven) mechanisms, previous stimuli or priming^49^ among others. As such, the quantitative comparison with the ground truth fixations of the NUS3D dataset, needed for a formal evaluation of the model, does not capture the system’s true merit, that is, the robust selection of nearby objects in dynamic environments within 170 ms.

Although the saliency maps from the model can thus not be directly compared with fixation maps, the model still reasonably represents interesting regions of the scene. In general, the evProtoDepth model shows a more localised response to near-objects when compared with the fbProtoDepth model (see Fig. 3). We believe this is mainly due to the sequential nature of processing in the event-based model. The simulated events used in this case first extract contrast information from the scene. Subsequently, only the depth information at event locations is used to inform the proto-object model. Therefore, the evProtoDepth model, having only one channel (depth), inherently prioritises the closer objects. In contrast, the frame-based model combines information from multiple channels (depth, colour opponency, intensity, orientations) at the latter stage of the pipeline, causing multiple features to contribute to predict the salient regions. The combination of cues from multiple channels produces a more dispersed overall saliency response. This may also lead to the fbProtoDepth model selecting objects with high contrast edges possibly located far away from the camera. We believe that prioritisation of close objects, at the cost of decreased attention to distant objects, is of high importance for a robotic agent because of its need for interactions with physical objects. Nevertheless, on the long run information from different sub-modalities and from different distances needs to be integrated and weighted appropriately. The proposed model acts as a first milestone towards more complex robotic attentive systems that can include other important cues such as contrast, motion, colour and orientation. Furthermore, in future developments, such an entirely data-driven system could be enriched with top-down mechanisms, enabling the machine to switch priorities between extracted features depending on the robot’s behavioural goals.

Additionally, in a more complete robotic pipeline, the saliency map could drive the robot’s gaze in a more natural way. In fact, humans continuously gaze in order to bring the region of interest onto the fovea. In another work^50^, we proposed an eccentricity model for sub-sampling the input visual space similar to that performed by a biological retina. In brief, the periphery of the field of view has coarser resolution than the middle (fovea). Combining such a model with an attentive system could be used in a pipeline that exploits saliency to drive the robot’s eyes towards the most interesting regions, thereby giving salient regions a higher sensory resolution required for higher-level processing. This mechanism would both bestow the robot with a natural behaviour similar to that found in biology, and would also lead to savings in computational resources, since only salient regions are processed at the full resolution.

This work attempts to bridge the gap between biologically plausible saliency models and bio-inspired hardware. We demonstrated the model running online on a humanoid robot in different scenarios proving how event-driven cameras are well-suited for saliency detection in embodied agents. Stereo event cameras allowed the easy extraction of moving edges, solving the correspondence problem using precise spiking times, and the removal of layers of processing from the fbProtoDepth. The long term goal would be to implement such a complex algorithm onto neuromorphic specialised platforms^51,52^ to better exploit the event-driven pipeline aiming to further decrease the computational cost of the system in terms of latency and power consumption.

## Methods

Traditional frame-based cameras generate frames synchronously at a fixed rate regardless of changes in the scene. For this reason the output contains great amounts of redundant data, especially in case of static scenes. Unlike regular cameras, event-driven sensors overcome the data redundancy providing data-driven output. This is particularly suitable for online robotic applications^53–56^ given the need for low latency and high speed^57,58^. Event-driven cameras react to illumination changes at the pixel level, generating an asynchronous stream of events. Each event is defined as a tuple (*x*, *y*, *p*, *t*), where *x* and *y* are the spatial coordinates of the instantaneously active pixel, *p* the polarity bit encoding the direction of the illumination change (dark-to-light or light-to-dark), and *t* timestamp when the event occurs at microsecond resolution. An example of an event stream plotted in spatio-temporal coordinates is shown in the middle column of Fig. 1.

In this study, we combine evProto^16^, a previously developed event-based model for attentional selection with fbProtoDepth, a frame-based proto-object model that incorporates depth information^26^ to develop the first version of an event-driven based saliency model in 3D which we call evProtoDepth. The current model uses depth as the primary channel for computing saliency. Depth perception is introduced *via* scene disparity extracted from stereo event cameras. Disparity is extracted using an asynchronous event-based bio-inspired cooperative neural network able to solve the correspondence problem^24^ in a scenario with multiple objects. The disparity-encoded events from the disparity extractor are accumulated into non-overlapping disparity frames of 100 ms duration, and are processed by the Border Ownership and Grouping Pyramids mechanisms in evProto to form proto-objects in the disparity map. An overview of the processing pipeline of the evProtoDepth model is presented in Supplementary Fig. S1. We designed and implemented the model for real-time usage on the iCub robot.

### Event-driven disparity extraction

In robotics, depth cues are important to select reachable objects upon which the robot can act, in addition to providing input for other tasks. The fbProtoDepth model uses depth from an RGB-D sensor. In order to implement a fully bio-inspired pipeline, we use disparity estimation techniques using stereo event-driven cameras as input for the evProtoDepth model. Binocular disparity of a 3D point relays information about its distance from the plane of fixation, but suffers from the problem of false correspondences. It is now widely accepted that mammalian brains solve this problem relying on a competitive process in disparity-sensitive neuron populations to encode and detect horizontal disparity^59^. Neurons compete with each other to represent the disparity of the scene, by removing false matching to reach a global solution. In particular, a disparity Cooperative Network^60^ employs correspondence between a stereo event-pair, and it imposes disparity uniqueness and continuity conditions to construct a map representing the level of belief/confidence of corresponding points.

Asynchronous cooperative matching processing is well-suited to exploit the output of event-driven cameras since the precise timing of event generation can be used to find correspondences efficiently at pixel-level without the need for patch or feature-based matching. This can produce disparity maps that can adapt to a dynamic input scene in real-time. Event-based cooperative matching algorithms have been efficiently implemented on neuromorphic platforms using Spiking Neural Networks (SNN)^61,62^ as well as on traditional computing platforms^24,63^. Although specialised neuromorphic hardware^51,52^ is well-adapted for spike-based computation due to its low latency and power consumption, these new generation devices have difficulty handling networks with hundreds of thousands of neurons working in real-time on robotic platforms which demand robustness. This model implements an array-based representation of a Spiking Neural Network (SNN) based on an Event-based Cooperative Stereo Matching^24^, similar to the SNN proposed by Osswald^61^. Our work implemented a real-time version of this algorithm on a standard CPU, prioritising its ease of deployment on the iCub and integration with the proto-object model over power consumption and efficiency afforded by neuromorphic hardware. It uses a 3D voxel-grid in $x-y-d$ space (*d*=disparity), called an *activity map*, which is updated asynchronously with each incoming event. Each element (cell) of this array represents a computational neuron in the SNN, which spikes during simultaneous triggering of events in the left and right camera. To ensure that temporally close events have higher probability to correspond to each other, a simplified version of the Leaky Integrate and Fire (LIF) model^64^ is used to model the internal dynamics of each activity cell.

The output disparity value *d* for each pixel corresponds to the layer with the highest activity (belief) for that pixel. Each incoming rectified event affects multiple cells in the *activity map* through excitatory and inhibitory connections. The excitatory connections enforce continuity constraints by ensuring that neighbouring pixels have similar disparity values, implementing the prior that most surfaces in the 3D environment are continuous and smooth. The inhibitory connections enforce uniqueness constraints by suppressing false correspondences between stereo-pairs along the line of sight. They ensure that each pixel is assigned only one disparity value. The strength of interaction is determined by the time difference between successive interactions, such that a cell affected by multiple events in close temporal proximity will be highly active. The activity generated by each incoming event on a particular voxel is inversely proportional to how far in the past that voxel was last affected. After several cycles of excitation and inhibition within the activity map, a *disparity event* is generated by the network by associating the incoming event with the disparity value of the layer that has the highest activity. The output of the network consists of estimates of the disparities of all events and collects them in a single channel of disparity events $E_d$ in the reference view of the left camera frame. With the event-based cooperative matching algorithm, we gain improvements over frame-based processing algorithms in terms of processing time at the cost of accuracy of disparity. The resulting disparity maps are sparse and prone to noise, especially when the input event throughput is high, e.g. when the camera moves in a textured scene. However, this suits our needs as the downstream proto-object saliency model acts as a filter that suppresses noise in the disparity maps while selecting the nearest object (e.g. Fig. 7). A schematic illustration of the network architecture is shown in Supplementary Fig. S2. Further details about the disparity extraction algorithm is provided in the supplementary material.

### Proto-object based saliency with depth information

Variations and extensions of proto-object saliency models using frame-based cameras include the addition of addtional features including motion^65^, texture^66^ and depth^26,27^ (we call the latter fbProtoDepth). Each information channel is separately processed by a “grouping” layer, that represents proto-objects in the final saliency map combining all channels.

A previous event-driven implementation of the proto-object model^16^ (evProto) focused on the use of event-driven cameras. The model exploits the inherent edge extraction capabilities of event-driven cameras, allowing it to omit the Gabor and center-surround filtering of the original frame-based model^11^. The output from the cameras is directly fed into the Border Ownership layer and processed in the same way as in the original version, detecting salient regions of the scene with a latency of $\approx 170 \; \hbox {ms}$ every time there is a change in the scene.

The fbProtoDepth model^26^ uses intensity, orientation, colour opponency and depth channels in parallel to compute saliency. In the evProtoDepth model, we implemented a single depth information channel, in the form of disparity-weighted event frames, fed into the grouping layer of the evProto model. The disparity of each individual event (based on the input from both cameras) is computed using a cooperative network model. Each output *disparity event* $E_d$ contains information about the pixel (*x*, *y*), generation time *ts* and disparity estimate *d* of the corresponding visual stimulus. Disparity events arriving within a time window $\delta t$ are accumulated in a disparity frame *D*(*x*, *y*, *t*). Each of its pixels stores the disparity value *d* of the latest disparity event $E_d$ emitted within that temporal window $(t-\delta t, t)$ at pixel (*x*, *y*). The length of the time-window is selected based on the desired sparseness of the disparity map fed into the grouping layer of the evProto model. The disparity frames are subsequently normalised within [0, 1] and passed onto the evProto model. While the input map in the original evProto model accounted for the presence of edges, our implementation extends this representation by also encoding the depth of each edge. The key components of the evProto model^16^ and proposed evProtoDepth models are shown in Supplementary Fig. S1.

### Consent statement

Informed consent has been obtained from the respective individual to publish images (Fig. 7 and Supplementary Fig. S10) in an online open access publication.

## Supplementary Information

Supplementary Information.

## Data Availability

The datasets generated and analysed during the current study are available from the corresponding authors on reasonable request.
