# Supplementary material for: Event-driven proto-object based saliency in 3D space to attract a robot’s attention
Source: Sci Rep. 2022 May 10;12:7645. doi: 10.1038/s41598-022-11723-6 (PMC9090933; doi:10.1038/s41598-022-11723-6)
Supplement: Supplementary file 1 — Supplementary Information. [file 41598_2022_11723_MOESM1_ESM.pdf]

Event-driven Proto-object based saliency in 3D space to attract a robot's attention –Supplementary Material–

Suman Ghosh[1,2,+], Giulia D'Angelo[1,3,+], Arren Glover[1], Massimiliano Iacono[1], Ernst Niebur[4], and Chiara Bartolozzi[1,*]

[1]Istituto Italiano di Tecnologia, Event Driven Perception for Robotics, Genoa, 16163, Italy
[2]S.G. is now at Technische Universität Berlin, Electrical Engineering and Computer Science, Berlin, 10623, Germany
[3]The University of Manchester, Department of Computer Science, Manchester, M13 9PL, UK
[4]Johns Hopkins University, Mind/Brain Institute, Baltimore, 21218, Maryland, USA
[+]these authors contributed equally to this work
[*]Correspondence: chiara.bartolozzi@iit.it

Multimedia Material

A supplementary video summarizing proposed methodology and results can be found at https://zenodo.org/record/5091539/files/evProtoDepth.mp4?download=1

Event-based proto-object model structure and parameters

Parameter	Value
R_0	10
ρ	0.2
Pyramid levels	5
Orientations	0°, 45°, 90°, 135°

Supplementary table S1. Parameters used in the 2D proto-object model[1] and the proposed evProtoDepth model. R_0 is the radius of the filter, ρ determines the arc length of active pixels in the kernel allowing to change the convexity of the kernel, *Pyramid levels* the number of scales and the *Orientations* of the Von Mises filters.

A schematic diagram of the event-driven proto-object models in both 2D (evProto[1]) and 3D (our proposed evProtoDepth) is depicted in Fig S1. The parameters used in the proto-object model are listed in Table S1.

Disparity computation model

We computed disparity of the scene on a per-event basis using a cooperative network that employs time correspondence between a stereo event-pair and imposes the disparity uniqueness and continuity conditions proposed by Marr and Poggio[3] to model a correspondence belief map. Inspired from Firouzi et al.[2], we used a 3D array-based representation of the network, called an activity map C, which gets updated with each incoming input event. Each element (cell) of this array abstracts a computational neuron in the Spiking Neural Network, where each correspondence neuron spikes during simultaneous triggering of events in its associated left and right pixels. At any time instant, the current state of the activity map represents the disparity of the present input scene. Each incoming event from the left or right camera gets processed asynchronously in the network without any explicit synchronization between them. A schematic diagram of the cooperative network used is shown in Fig S2.

Each incoming event was remapped to its pixels coordinate using pre-calibrated stereo camera parameters to ensure that the corresponding left and right events have the same y coordinates. The stereo correspondence search thus gets simplified to a single row scan due to the inherent epipolar constraints. We define disparity as $d = x_l - x_r$,

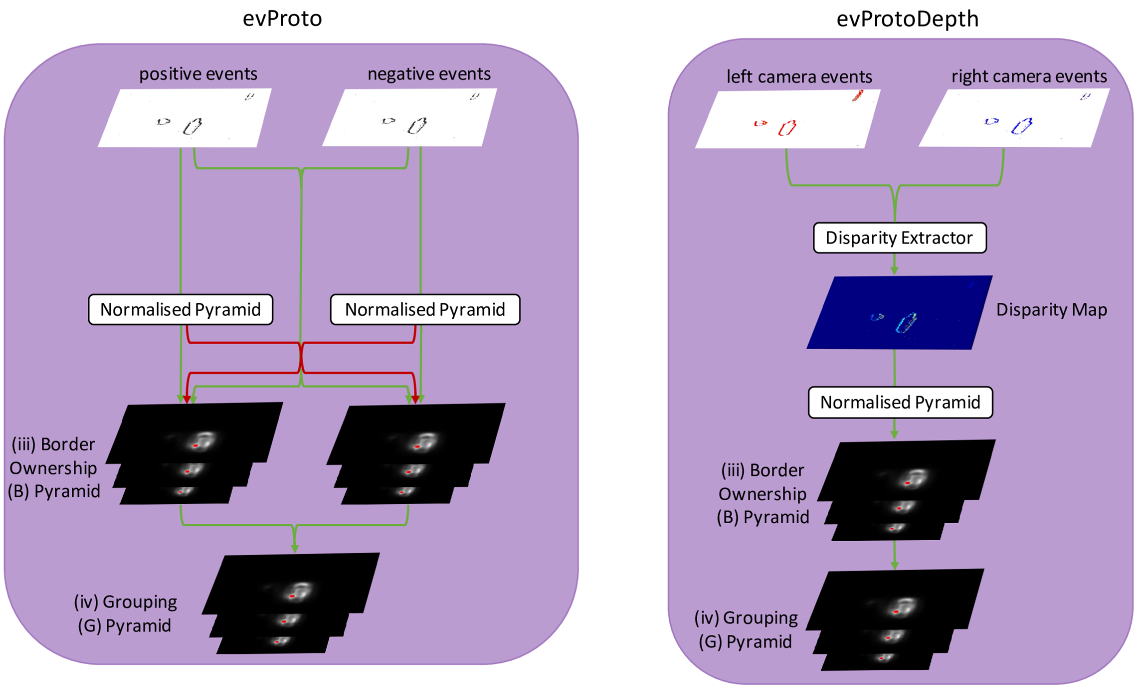

Supplementary figure S1. Schematic representation of the evProto (left) and evProtoDepth (right) models. Both models show size invariance due to the 5 levels of the Normalised Pyramid. In the evProto model, positive and negative events go into the two core stages of the model: Border Ownership and Grouping. In the case of the evProtoDepth, the binocular events are fed into the disparity Extractor producing a Disparity Map. The Border Ownership takes in input the normalised outcome from the Pyramid fed with the disparity events.

where x_l and x_r are rectified events from the left and right cameras respectively. Since the rectified image planes are parallel, we only have positive disparities. Due to the asymmetric nature of the disparity computation, the left and right events are processed non-identically. We present here the relevant computations performed on an incoming left event. The corresponding operations to be performed on a right event then follows by changing the signs for disparity equations as the direction of matching switches. Furthermore, the activity map and hence the disparity map, is represented with the reference of the left camera frame. It is a matter of arbitrary choice, and each pixel coordinate only needs to be horizontally shifted by its computed disparity when a right reference frame is used.

We consider an input rectified event $E_l = (P_l, t_l)$ that is generated from the left sensor at time t_l and pixel location $P_l = (x_l, y_l)$. The cooperative network computes the best disparity value for this event. Each activity cell C_{x_l,y_l,d_k} encodes the belief of the system about whether x_l from the left event and $x_r = x_l - d_k$ from the right event are true stereo correspondences, in the form of activity. Consequently, each cell encodes the validity of the disparity value d_k for event E_l. The size of the matrix is thus $M \times N \times d_{max}$, where $M \times N$ is the sensor dimension.

Due to epipolar constraints, the set of possible corresponding pixels in the right image are:

$$S_l = \{(x_r, y_r) \mid x_l - d_{max} \leq x_r \leq x_l, y_r = y_l\} \tag{1}$$

where d_{max} is an algorithmic parameter that determines the maximum detectable disparity. Each element of this correspondence set represents a layer d_k in the activity map for P_l. Therefore, a cell C_{x_l,y_l,d_k} represents a candidate correspondence in S_l. For a single incoming event, we thus compute the activity related to each candidate correspondence in S_l. The cooperative network evaluates the legitimacy of each candidate in S_l for its true correspondence to P_l, using a Winner-Takes-All mechanism. The winner disparity value d_{WTA}, representing the d_k layer with the maximum activity value (above a predefined threshold θ), is the final computed disparity for the input event E_l.

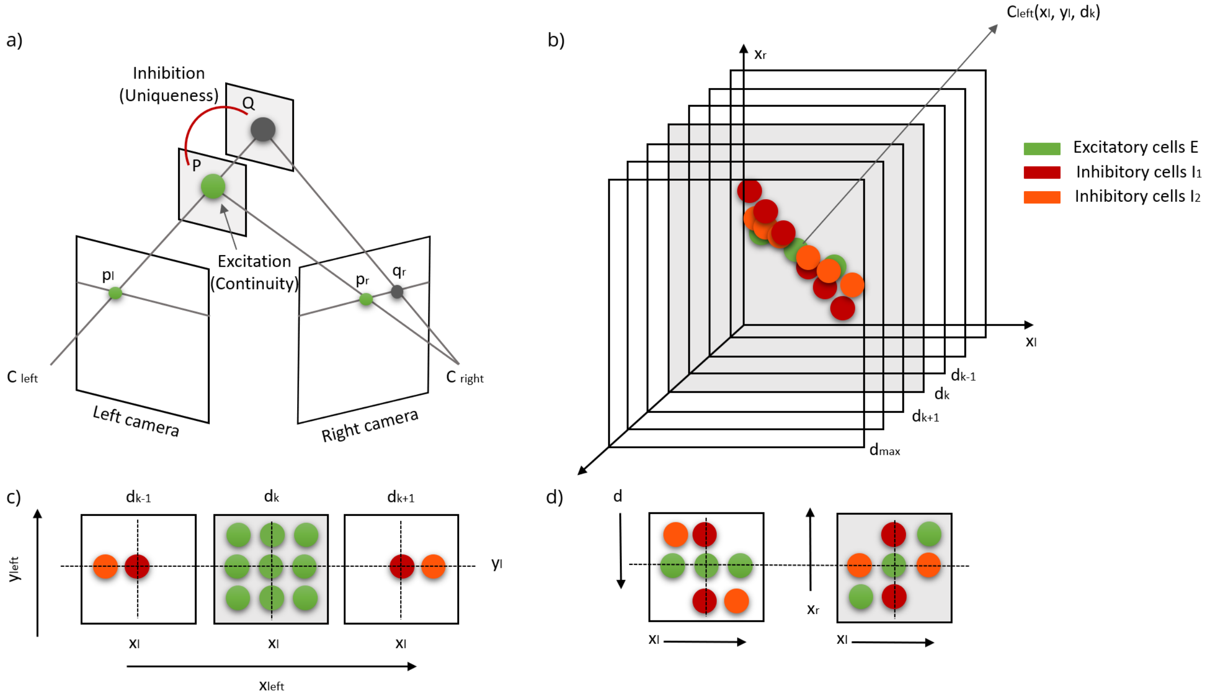

Supplementary figure S2. Asynchronous Cooperative Stereo Network for event-based disparity estimation[2]. a) Representation of the stereopsis problem describing continuity and uniqueness constraints b) three-dimensional view of the Excitation and Inhibition Network. The axes refer to the horizontal coordinates of the two cameras and the disparity c), d) Excitation and Inhibition Network on a two-dimensional plane: c)x_l, y_l axes and d) *disparity*, x_l & x_l, x_r axes.

$$d_{WTA}(E_l) = \underset{d_k}{\arg\max}\left\{C_{x_l,y_l,d_k} \mid C_{x_l,y_l,d_k} \geq \theta\right\} \tag{2}$$

The activity for each cell is computed using time-weighted excitatory and inhibitory connections from previously activated cells in the network. These connections are determined according to the continuity and uniqueness disparity constraints respectively.

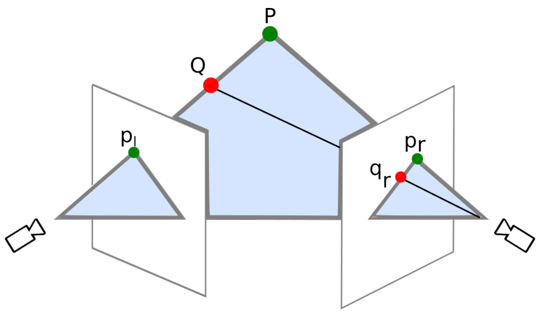

Supplementary figure S3. Event correspondence

Excitatory connections

To enforce the within-disparity continuity constraint, cells in the same disparity layer surrounding the firing pixel should potentiate each other. This leads to more contiguous regions in the disparity map based on the reasoning

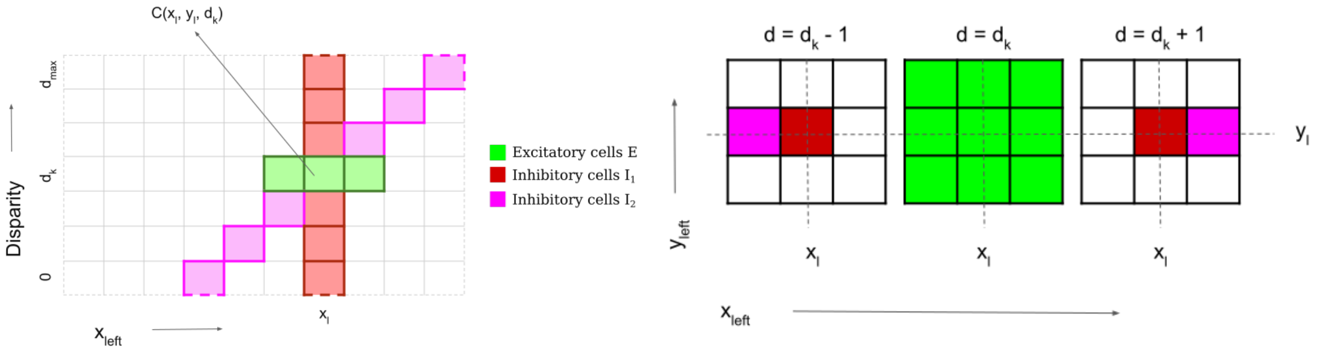

Supplementary figure S4. C_{x_l,y_l,d_k} depicted in cross-sectional views of the activity map, along with its excitatory and inhibitory sets, given by equations 3, 4 and 5, with parameters $d_{max} = 6$ and $r = 1$. Top: cross-sectional view at vertical layer y_l; Bottom: cross-sectional views at disparity layers $d_k - 1$, d_k and $d_k + 1$.

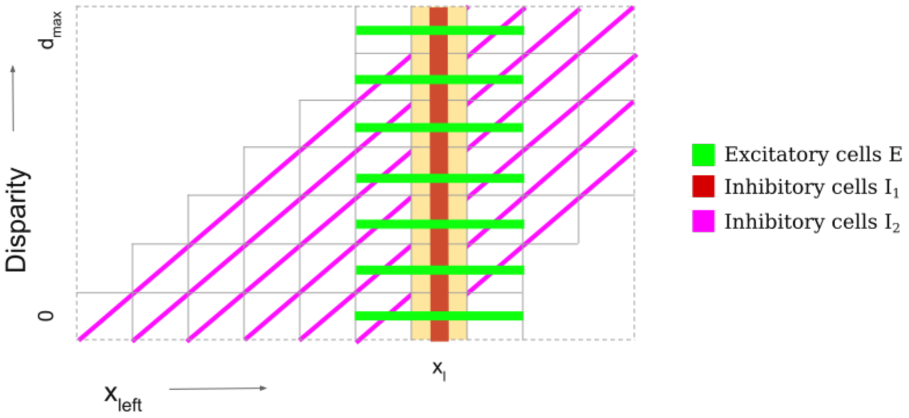

Supplementary figure S5. Cross-section of activity map C at layer y_l, showing all excitatory and inhibitory sets for an input left event e_l at pixel $P_l = (x_l, y_l)$, with parameters $d_{max} = 6$ and $r = 1$. Each yellow element in the column x_l represents an activity cell $C_{x_l,y_l,d}$. The lines of the excitatory and inhibitory sets for each cell intersect at its center. Inhibition set I_1 is nearly same (all cells along the x_l column except the cell in focus) for all candidate correspondences.

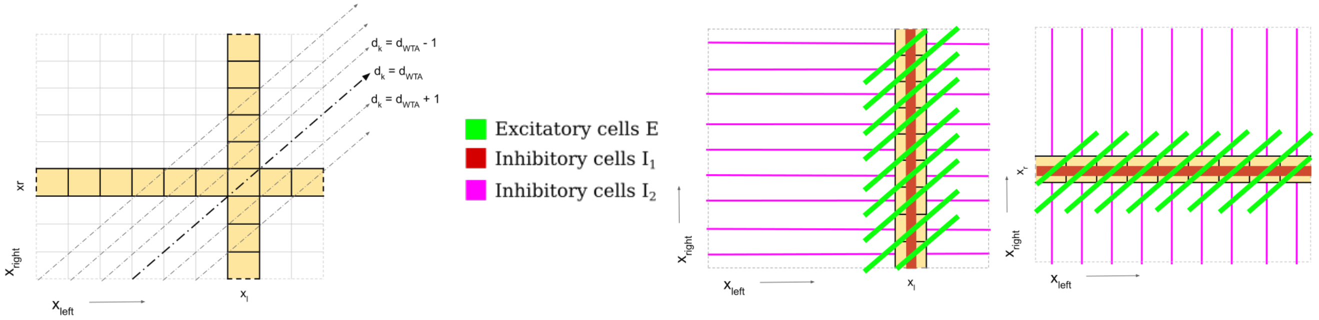

Supplementary figure S6. Network computations for matching left and right event pair with pixel coordinates (x_l, y) and (x_r, y) respectively, with parameters $d_{max} = 6$ and $r = 1$. Top-left: Representation of estimated true disparity d_{WTA} in left-right x-coordinate correspondence map; Bottom-left: All network excitations and inhibitions for input left event at (x_l, y); Bottom-right: All network excitations and inhibitions for input right event at (x_r, y).

that pixels in the close neighbourhood should possess similar disparity values. Events generated by extended objects in the spatial and temporal vicinity thus lead to more accurate disparity values. Therefore, for each element

in S_l, the set of excitatory connections are defined as:

$$E(C_{x_l,y_l,d_k}) = \{C_{x',y',d_k} \mid |x' - x_l| \leq r, |y' - y_l| \leq r\} \quad (3)$$

where r is the size of the neighborhood which we consider for excitation.

Inhibitory connections

To enforce the cross-disparity uniqueness conditions, cells in the same epipolar line contributing to other disparities should inhibit the current disparity belief evaluation. For each candidate correspondence in S_l, we use two kinds of inhibitory connections in the network.

The first set of inhibitory connections are defined as

$$I_1(C_{x_l,y_l,d_k}) = \{C_{x',y',d} \mid 0 \leq d \leq d_{max}, d \neq d_k, x' = x_l, y' = y_l\} \quad (4)$$

This set of inhibitory cells topologically represent correspondence between the current cell C_{x_l,y_l,d_k} and all other pixels lying on the conjugate epipolar line of P_l in the right image. This means that for a left event, a candidate disparity that has already been assigned a high belief by the network, inhibits all other possible disparities for that event. Lateral inhibition like this is present throughout the human vision system[4]. It helps to reduce false positive matches in noisy environments. A false positive matching scenario is depicted in figure S3 – if p_l on the left image is a retinal projection of the point object P, there are two possible matches in the right retina, p_r or q_r. However, since just one of the candidates can be chosen, the correspondence p_l - p_r should inhibit p_l - q_r so that it does not lead to the false positive 3D point Q.

The second set of inhibitory connections is defined as:

$$I_2(C_{x_l,y_l,d_k}) = \{C_{x',y',d} \mid 0 \leq d \leq d_{max}, d \neq d_k, x' = x_l - d_k + d, y' = y_l\} \quad (5)$$

This layer of inhibition, as proposed in[2], is used to further reduce false matches by enforcing that a candidate right pixel should contribute to only one stereo correspondence. For each disparity level d_k, it may happen that the corresponding right pixel $(x_r = x_l - d_k, y_r = y_l)$ in S_l has already contributed to a stereo match in an earlier iteration of the algorithm. We thus inhibit the current correspondence belief C_{x_l,y_l,d_k}, using the summation of cell activities (x_r, y_r) might have contributed to. Since the C is formulated with reference to the left image frame, x_r is actually represented as $x' = x_r + d$ in the activity map. This extra layer of inhibition resolves disparity ambiguity in the scene caused by multiple bodies, therefore producing more precise disparity maps.

Figure S4 illustrates the excitatory set E (in green), as well as inhibitory sets I_1 (in red) and I_2 (in magenta) for a single activity cell C_{x_l,y_l,d_k}. Both (x_{left}-disparity) and (x_{left}-y_{left}) cross-sections of a section of the 3D activity map C are shown. These sets are computed for each candidate correspondence element in S_l. Thus, computations for an input left event e_l with pixel coordinates $P_l = (x_l, y_l)$ are affected by multiple excitatory and inhibitory sets as depicted in figure S5.

Temporal Correspondence for Activity computation

Events are triggered by the ATIS cameras whenever there is change is illumination in the input scene. Thus, events originating from the left and right cameras due to the same source are generated around the same time. The temporal proximity of corresponding events thus helps in better stereo matching. Ideally, temporal coincidence should represent pixel correspondence on an event level. However, input stimuli with noise and multiple extended objects generate a lot of events around the same time, thus the timing information encoded in the events are not perfect due to jitter in latency of the acquisition system from the left and right cameras. Furthermore, the order of generated events from each camera may also be incoherent. The cooperative network we use ensures that the disparity estimation works even when timing information is not precise.

To ensure that temporally close events have higher probability to correspond to each other, a simplified abstraction of Leaky Integrate and Fire (LIF) model[5] is used to model the internal dynamics of each activity cell. An activation time is maintained for each cell in the activity map. Every time a cell in the network $C_{x,y,d}$ gets activated due to an incoming input event, we update its activation time $t_{x,y,d}$. We use a temporal kernel W that weights the contribution of each interacting cell (excitatory and inhibitory) based on how far ago in time they were activated, with respect to the current event time t_l. It is defined as follows:

$$W_{x,y,d}^{t_l} = \frac{1}{1 + \beta(t_l - t_{x,y,d})} \tag{6}$$

where t_l is the current activation time, and $t_{x,y,d}$ is the time when the activity of cell $C_{x,y,d}$ was last activated.

Therefore, for an incoming left event E_l, activities of all network cells, each corresponding to a disparity layer, are computed in a single pass and stored in temporary one-dimensional array. Using the temporal kernel W, excitation set E, and inhibition sets I_1 and I_2, the activity of each cell C_{x_l,y_l,d_k} is computed as:

$$C_{x_l,y_l,d_k} = \sigma\left(\sum_{x',y',d' \epsilon E} W_{x',y',d'}^{t_l} C_{x',y',d'} - \alpha \sum_{x',y',d' \epsilon I_1 \cup I_2} W_{x',y',d'}^{t_l} C_{x',y',d'} \right) \tag{7}$$

where the sigmoid function $\sigma(k) = \frac{1}{1+e^{-k}}$ is used to normalize the activity to values between 0 and 1. Using equation 2, we estimate the disparity value for E_l. The activity values and their respective trigger times are finally updated inside the network in a single pass.

When two events e_l and e_r from the left and right cameras are generated close in time, the network computes their disparity as $d_{WTA} = x_l - x_r$, where x_l and x_r are respective horizontal pixel coordinates of the events. This is ensured by appropriately weighting the inhibitory and excitatory connections in the network, using the parameter α. Figure S6 illustrates the computations performed by the network for these two corresponding events. In this figure, all connections are plotted in the domain of x_{left} - x_{right} correspondence maps for symmetrical representation. The candidate matching cells for both events (shown in yellow) lie along rows and columns of the correspondence map. Layers of constant disparity are shown with dashed arrows. The figure explains how the cooperative network excites disparity in neighbouring regions, and inhibits disparities along epipolar lines. However, for ease of implementation, we map all computations in the x - disparity domain, like in figure S5. This leads to efficient traversal across disparity layers represented by straight lines along contiguous 1-D array elements.

Parameter	Value
r	3
θ	0.4
α	0.3
β	0.0001
d_{max}	45

Supplementary table S2. Parameters used for disparity computation using the asynchronous cooperative network. Excitatory neighborhood r tunes the smoothness of the disparity maps. Activation function threshold θ can be used to adjust the desired trade-off between noise and sparsity of output – high values will filter out noisy predictions with low activity but may also remove valid estimates. Inhibitory factor α tunes the strength of inhibition during cooperation. Slope of temporal correlation kernel β can adjust the temporal sensitivity of the cells to input events – higher value means faster dynamics and sharper temporal sensitivity to the upcoming events. The number of disparity levels d_{max} is used to modulate the precision of disparity estimation at the cost of increased computational overheads.

The empirically chosen parameters of disparity extractor are listed in Table S2.

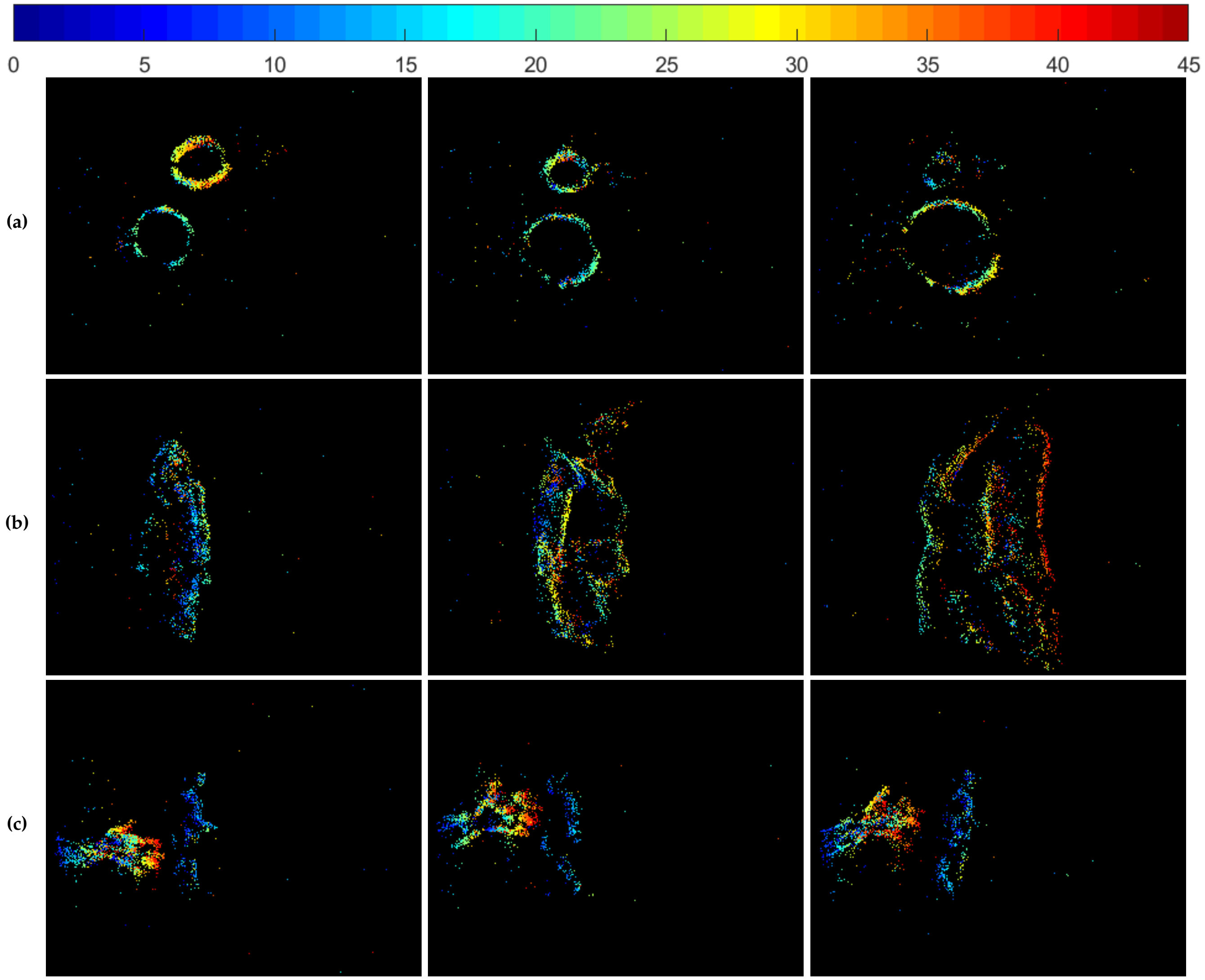

Supplementary figure S7. Estimated disparity for various instances of three sequences: (a) Two paddles of different sizes moving towards and away from the cameras, along the depth axis of the robot's cameras. Pixels where events occur during the 100ms time-window are colour-coded as per the computed disparity (depth). The object stimuli switch colours as their relative depth changes. (b) Same as (a) but for a person with at varying distances between 30 cm and 210 cm from the robot. (c) Scene showing two persons at different depths from the robot. Person 1 (mainly yellow and red pixes) is waving a hand at 30 cm depth. Person 2 (mainly blue) is walking horizontally across the scene at ≈210 cm depth. Pixel colours represent depth, as in (a) and (b), and remain constant for motion in the same depth plane.

Saliency Benchmarking with NUS-3D dataset

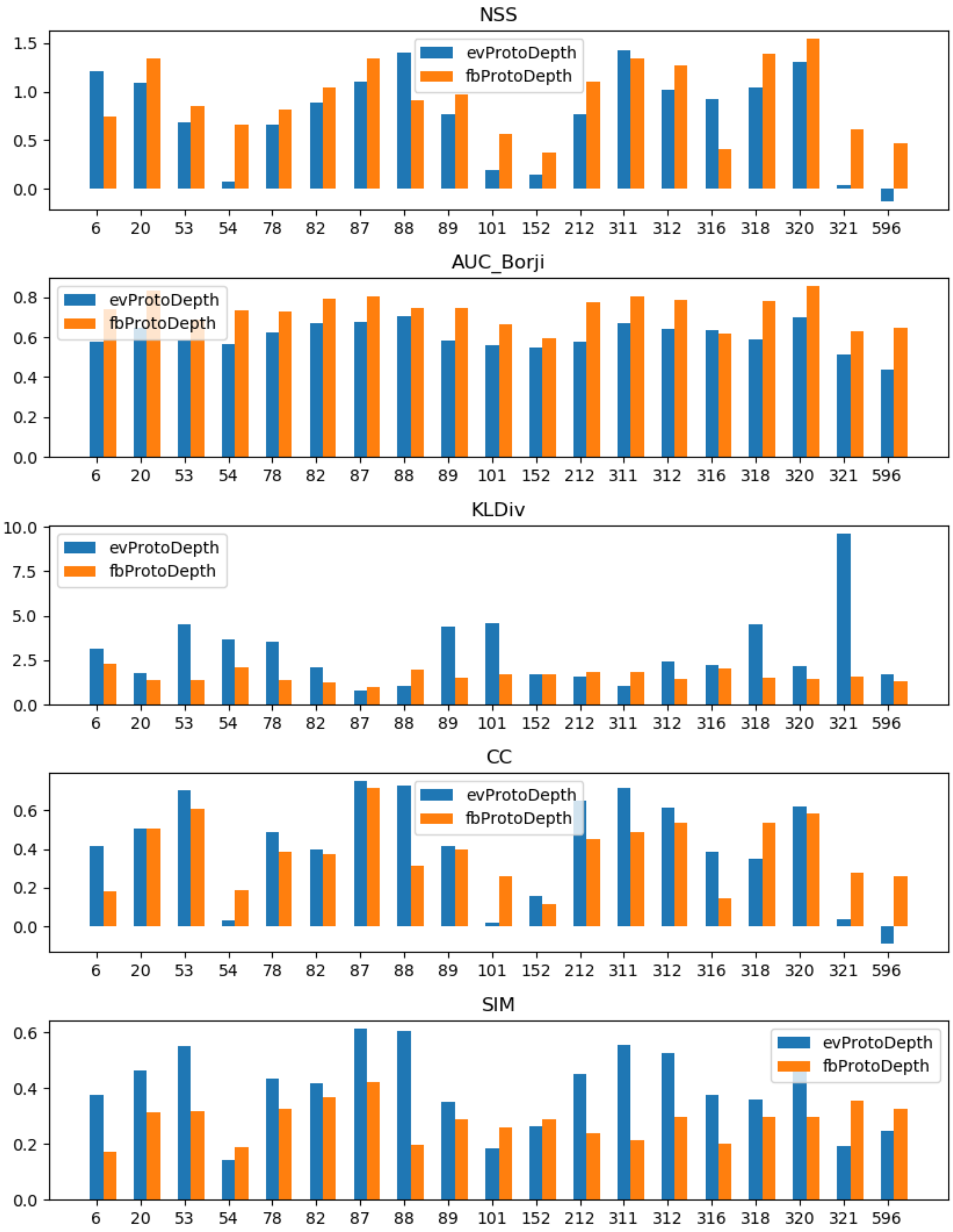

Supplementary figure S8. Quantitative evaluation of saliency maps generated by fbProtoDepth[6] and evProtoDepth using the MIT saliency metrics Normalized Scanpath Saliency (NSS), Area under the ROC Curve (AUC-Borji), Kullback-Leibler Divergence (KLDiv), Pearson's Correlation Coefficent (CC) and Similarity (SIM)[7–10] on a subset of the NUS3D dataset where human eyes were fixated is mostly on the nearest object of the scene. The subset comprises all cases among the NUS-3D dataset where the cross-correlation between the ground truth 3D fixation and inverse of ground truth depth ≥ 0.5. The x-axis depicts the image number as present in the NUS-3D dataset. For all metrics except KLDiv, larger value is better.

Image #	RGB image	Saliency Map: fbProtoDepth[6]	Saliency Metrics: fbProtoDepth[6]	Saliency Map: evProtoDepth	Saliency Metrics: evProtoDepth	Ground truth 3D fixations
156	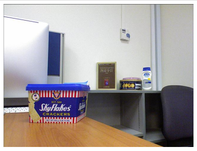	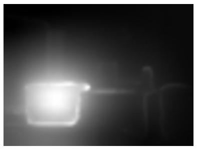	**NSS = 0.808**<br>**AUC-Borji = 0.708**<br>**KLDiv = 1.343**<br>**CC = 0.387**<br>**Sim = 0.362**	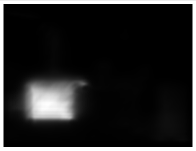	NSS = 0.655<br>AUC-Borji = 0.6<br>KLDiv = 3.215<br>CC = 0.364<br>Sim = 0.361	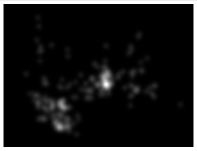
188	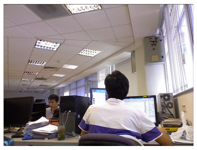	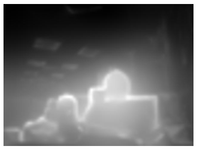	**NSS = 0.856**<br>**AUC-Borji = 0.74**<br>**KLDiv = 1.176**<br>**CC = 0.454**<br>**Sim = 0.384**	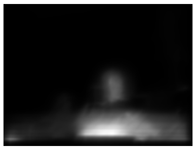	NSS = 0.357<br>AUC-Borji = 0.602<br>KLDiv = 5.086<br>CC = 0.116<br>Sim = 0.303	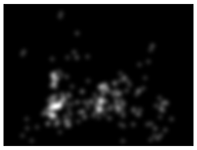
208	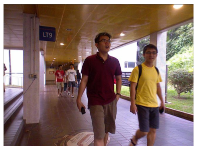	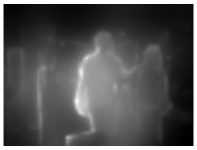	**NSS = 0.693**<br>**AUC-Borji = 0.704**<br>**KLDiv = 1.516**<br>**CC = 0.283**<br>**Sim = 0.285**	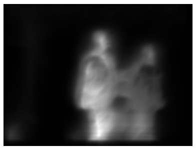	NSS = 0.339<br>AUC-Borji = 0.606<br>KLDiv = 3.622<br>CC = 0.171<br>Sim = 0.258	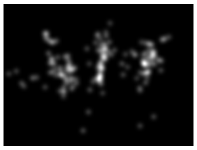
250	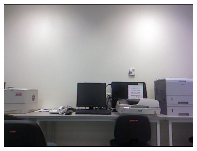	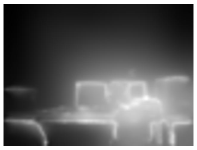	**NSS = 0.935**<br>**AUC-Borji = 0.74**<br>**KLDiv = 1.563**<br>**CC = 0.372**<br>**Sim = 0.285**	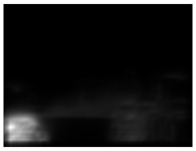	NSS = 0.15<br>AUC-Borji = 0.531<br>KLDiv = 3.3<br>CC = 0.054<br>Sim = 0.198	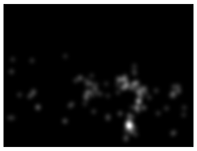
300	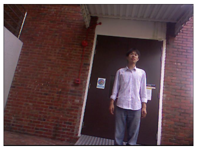	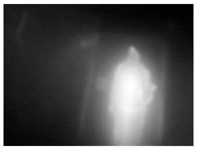	**NSS = 0.79**<br>**AUC-Borji = 0.705**<br>**KLDiv = 1.556**<br>**CC = 0.331**<br>**Sim = 0.292**	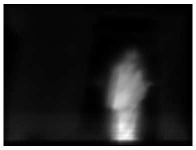	NSS = 0.482<br>AUC-Borji = 0.588<br>KLDiv = 2.319<br>CC = 0.172<br>Sim = 0.252	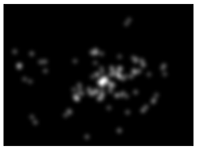
350	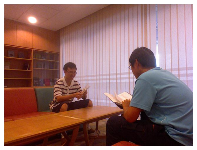	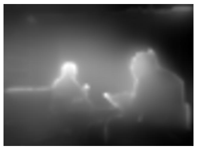	**NSS = 0.808**<br>**AUC-Borji = 0.713**<br>**KLDiv = 1.388**<br>**CC = 0.467**<br>**Sim = 0.318**	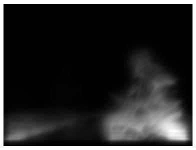	NSS = -0.031<br>AUC-Borji = 0.503<br>KLDiv = 6.676<br>CC = -0.051<br>Sim = 0.139	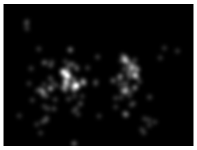
410	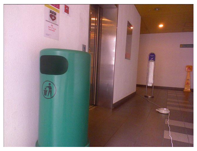	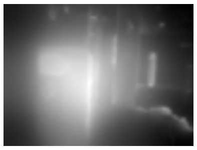	**NSS = 0.557**<br>**AUC-Borji = 0.657**<br>**KLDiv = 1.379**<br>**CC = 0.271**<br>**Sim = 0.325**	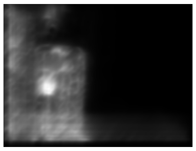	NSS = -0.057<br>AUC-Borji = 0.479<br>KLDiv = 12.357<br>CC = -0.037<br>Sim = 0.192	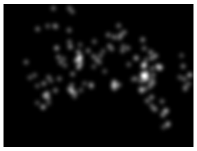
500	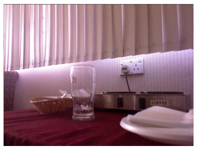	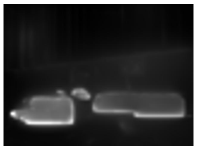	**NSS = 0.282**<br>**AUC-Borji = 0.636**<br>**KLDiv = 1.466**<br>**CC = 0.203**<br>**Sim = 0.312**	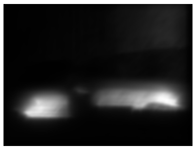	NSS = 0.236<br>AUC-Borji = 0.534<br>KLDiv = 2.157<br>CC = 0.15<br>Sim = 0.251	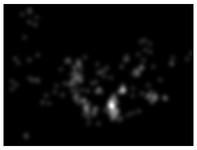
536	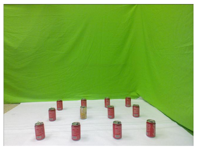	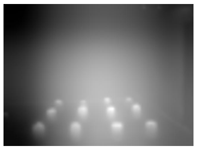	**NSS = 1.099**<br>**AUC-Borji = 0.81**<br>**KLDiv = 1.809**<br>**CC = 0.372**<br>**Sim = 0.248**	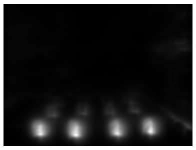	NSS = 0.602<br>AUC-Borji = 0.607<br>KLDiv = 2.335<br>CC = 0.13<br>Sim = 0.236	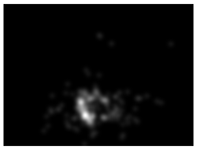
569	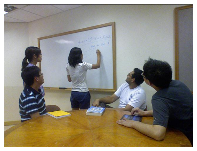	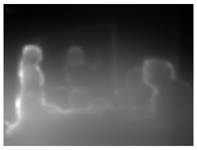	**NSS = 0.252**<br>**AUC-Borji = 0.565**<br>**KLDiv = 1.846**<br>**CC = 0.337**<br>**Sim = 0.251**	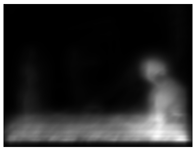	NSS = -0.218<br>AUC-Borji = 0.431<br>KLDiv = 6.576<br>CC = -0.138<br>Sim = 0.096	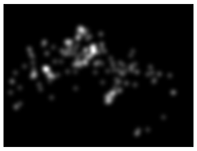

Supplementary figure S9. Example scenes from the publicly available NUS-3D dataset[11] where the frame-based fbProtoDepth model[6], which includes colour opponency as well as orientation information channels, quantitatively outperforms the event-based evProtoDepth model in all the MIT saliency benchmark metrics. Here, the ground truth fixations are not confined to the nearest "object" in the scene. Better values are bold-faced. The event-based model generates more localised saliency maps that are better at precisely selecting the nearest "object" since it mainly relies on depth and Gestalt cues from high contrast edges.

Robot experiments

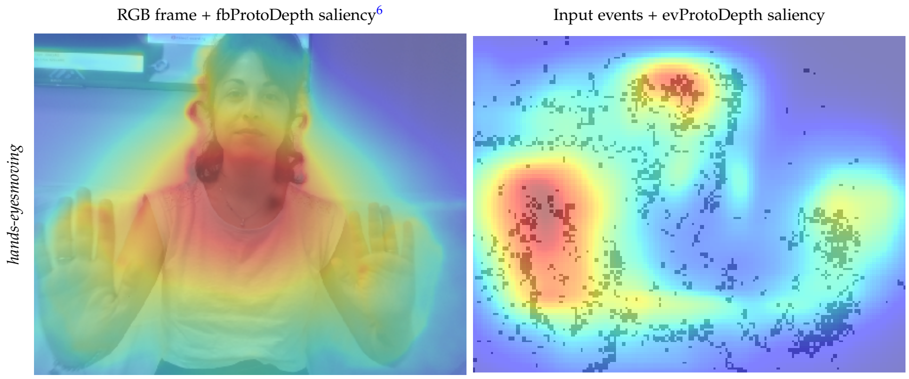

Supplementary figure S10. Comparison of saliency map selectivity between fbProtoDepth and evProtoDepth models running on the robot. Each plot shows the 2D histogram of accumulated saliency maps over all the frames of the *clutter* and *hands-eyesmoving* datasets. The frame-based model receives RGB and depth input from the RealSense camera, and the event-driven models receive input from the stereo event cameras. The two cameras have different field of views, and hence are not spatially aligned.

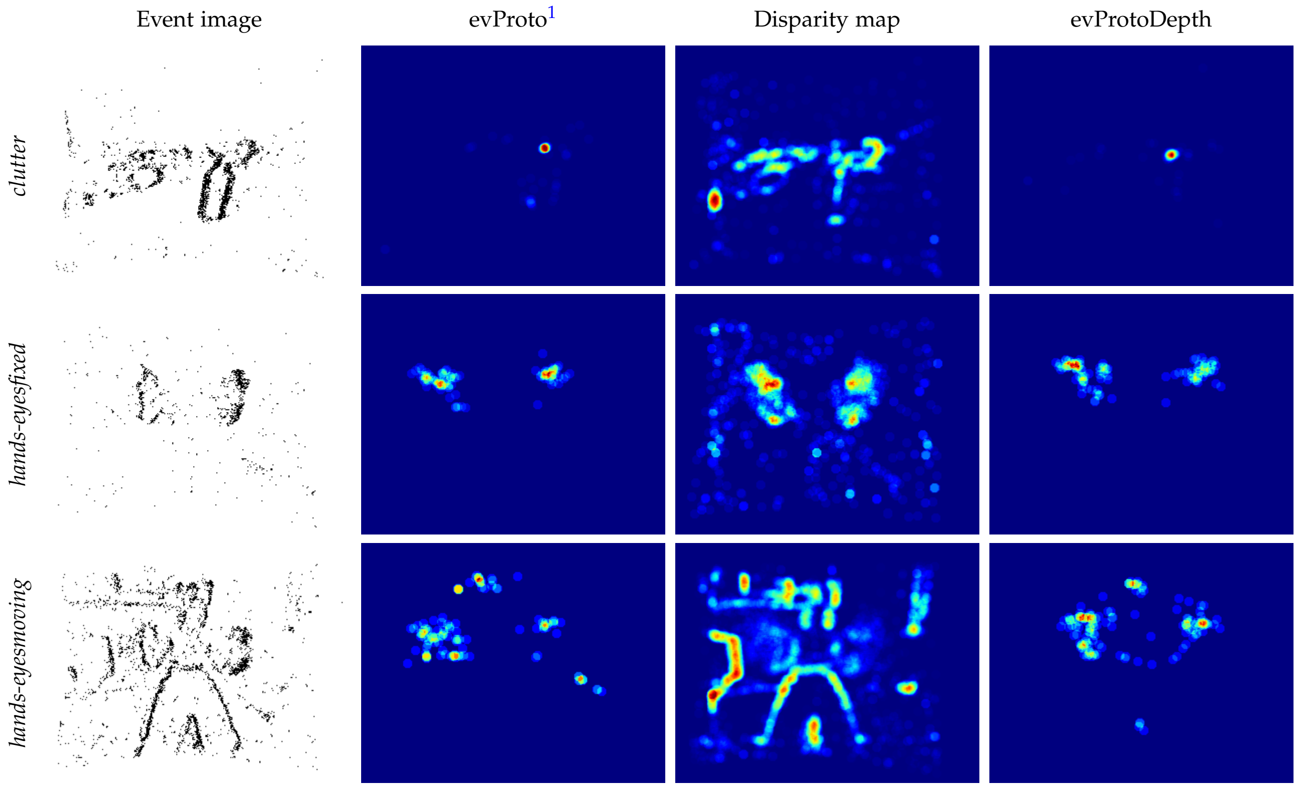

Supplementary figure S11. Contribution of both disparity and proto-object modelling in selecting the nearest (most salient) "object" on the *clutter* and *hands* datasets. Each plot depicts the 2D histogram of peak saliency pixel over all the frames of a dataset. For the disparity-only setup in Column 3, the peaks of the raw disparity map also includes non-salient regions of the scene, hence using disparity alone is not suitable for stable object selection. For the proto-object models in Column 2 and 4, the saliency peaks are more precisely concentrated at suitable proto-objects. The combination of disparity and proto-object modelling in evProtoDepth thus generates more robust and precise peak saliency locations than the individual 2D evProto model or the disparity map. While the disparity information improves selectivity of the saliency model, the proto-object model acts as filter to isolate high-level "proto-objects" from noisy depth maps.

References

1. Iacono, M. *et al.* Proto-object based saliency for event-driven cameras. In *IROS*, 805–812 (2019).
2. Firouzi, M. & Conradt, J. Asynchronous event-based cooperative stereo matching using neuromorphic silicon retinas. *Neural Process. Lett.* **43**, 311–326 (2016).
3. Marr, D. & Poggio, T. Cooperative computation of stereo disparity. *Science* **194**, 283–287 (1976).
4. Jerath, R., Cearley, S. M., Barnes, V. A. & Nixon-Shapiro, E. How lateral inhibition and fast retinogeniculo-cortical oscillations create vision: A new hypothesis. *Med. Hypotheses* **96**, 20 – 29, DOI: https://doi.org/10.1016/j.mehy.2016.09.015 (2016).
5. Gerstner, W., Kistler, W. M., Naud, R. & Paninski, L. *Neuronal Dynamics: From Single Neurons to Networks and Models of Cognition* (Cambridge University Press, 2014).
6. Hu, B., Kane-Jackson, R. & Niebur, E. A proto-object based saliency model in three-dimensional space. *Vis. research* **119**, 42–49 (2016).
7. Bylinskii, Z., Judd, T., Oliva, A., Torralba, A. & Durand, F. What do different evaluation metrics tell us about saliency models? *IEEE Transactions on Pattern Analysis Mach. Intell.* **41**, 740–757, DOI: 10.1109/TPAMI.2018.2815601 (2019).
8. Judd, T., Durand, F. & Torralba, A. A benchmark of computational models of saliency to predict human fixations. In *MIT Technical Report* (2012).
9. Borji, A., Sihite, D. N. & Itti, L. Quantitative analysis of human-model agreement in visual saliency modeling: A comparative study. *Image Process. IEEE Transactions on* **22**, 55–69 (2013).
10. Borji, A. & Itti, L. Cat2000: A large scale fixation dataset for boosting saliency research. *CVPR 2015 workshop on "Future Datasets"* (2015). ArXiv preprint arXiv:1505.03581.
11. Lang, C. *et al.* Depth matters: Influence of depth cues on visual saliency. In *Computer Vision–ECCV 2012*, 101–115 (Springer, 2012).
